# Fostering self-determination of bedside providers to promote active participation in rapid response events

**DOI:** 10.1080/10872981.2018.1551028

**Published:** 2018-11-30

**Authors:** Aarti C. Bavare, Jenilea K. Thomas, Lindsey M Gurganious, Natasha Afonso, Tessy A. Thomas, Satid Thammasitboon

**Affiliations:** a Baylor College of Medicine, Texas Children’s Hospital, Houston, Texas, USA; b Section of Critical Care Medicine, Department of Pediatrics, Baylor College of Medicine, Houston, Texas, USA; c Community Advance Practice Providers, Section of Critical Care Medicine, Department of Pediatrics, Baylor College of Medicine, Houston, Texas, USA; d Section of Palliative Care, Department of Pediatrics, Baylor College of Medicine, Houston, Texas, USA; e Section of Critical Care Medicine, Department of Pediatrics, Baylor College of Medicine, Houston, Texas, USA; f Section of Critical Care Medicine and Center for Research, Innovation and Scholarship in Medical Education, Department of Pediatrics, Baylor College of Medicine, Houston, Texas, USA

**Keywords:** Rapid Response teams, SDT: Self-Determination Theory, RR training, bedside providers

## Abstract

**Background**: Widespread implementation of rapid response (RR) systems positively impacts outcomes of clinically unstable hospitalized patients. Collaboration between bedside providers and specialized responding teams is crucial for effective functioning of RR system. Bedside, providers often harbor negative feelings about having to ‘call for help’ that could impact their active participation in RR.

**Objective**: The objective of the study is to enhance active participation of bedside providers in RR by fostering self-determination through targeted education.

**Design**: Needs assessment affirmed that bedside providers in our tertiary academic pediatric hospital felt loss of control over patient care, lack of competence, and disconnect from the RR team. We used the principles of autonomy, competence, and relatedness posited by the self-determination theory to guide the development, implementation, and evaluation of our educational program for bedside providers.

**Results**: Forty-two bedside providers participated in our program. Participants reported significant improvement in RR-related clinical knowledge. More importantly, there was significant enhancement in individual perceptions of autonomy (pre-mean: 2.12, post-mean: 4.4) competence (pre-mean: 2.15, post-mean: 4.4), and relatedness (pre-mean: 2.65, post-mean: 4.5) with RR (*p* < 0.01). The evaluation results for overall educational effectiveness showed a mean score of 4.69 ± 0.79. All scores were based on a 5-point Likert scale of 1: poor to 5: excellent. Educators noted good participant engagement. The program’s structure, evaluations, and data management were modified based on the feedback.

**Conclusions**: We successfully developed and implemented targeted educational program for bedside providers based on self-determination theory. The evaluations showed improvement in bedside providers’ clinical RR knowledge and perceptions of autonomy, competence, and relatedness following the training.

## Introduction

The Institute of Healthcare Improvement as a part of the ‘Save 100,000 Lives Campaign’ recommended widespread implementation of rapid response (RR) systems or medical emergency teams to propagate patient safety and improve clinical outcomes [1,2]. Numerous studies describe the positive impact of RR systems on patient outcomes, such as decrease in the rates of emergency transfers to intensive care units, critical deterioration (need for emergent ventilatory/hemodynamic support), cardiopulmonary arrests, and mortality [3–8]. Additionally, clinical providers describe RR-related positive impact on educational opportunities for caregivers and team morale [9,10].

Nonetheless, the bedside providers (nurses and physicians) who are usually the initial responders (IRs) report certain unintended affective consequences of RR events. Whereas the IRs usually feel relieved when help arrives in the form of specialized responders (SRs), they also perceive at times a criticism of their ongoing management that led to the need for RR and a burden to explain why the RR system was activated [11,12]. As the foundation of a RR system incorporates the concept of ‘failure to rescue’ [13,14], its activation can be perceived to result from deficiencies on the part of IRs related to the delayed, or nonrecognition, of clinical instability that necessitates a ‘rescue’ of the patient. Other affective consequences of the RR system reported in the literature include increased tension between nurses and physicians, excessive burden on providers, and decreased autonomy for trainees [10,15].

Adverse feelings harbored by IRs related to RR events can compromise timely activation of response, delay initiation of proper care, and result in harm to the patient [16,17]. RR training programs offered for specialized teams have a positive influence on the confidence and competence of these teams during RR events [18]. Little is documented in the literature about a similar training program for IRs, despite the universal expectation that they perform the important tasks of early detection of clinical instability and timely activation of response. To fill the need for RR education for IRs, we developed an educational program titled ‘Recognize-React-Teamwork: RRT Workshop.’ The workshop features were based on the components of self-determination theory (SDT). This theory describes that learning conditions that foster enhancement of learners’ autonomy, competence, and relatedness positively drive learners’ motivation to engage and persist in learning activities [19]. The overarching goal of our program was to educate pediatric bedside providers with knowledge, skills, and attitudes that would enhance their competency and satisfaction with performance during a RR event and promote their active participation in future RRs. We describe here the development, implementation, and preliminary evaluation of a theory-informed, educational program delivered in a workshop format.

## Materials and methods

The Institutional Review Board of Baylor College of Medicine reviewed and approved the project. Detailed needs assessment and framework conceptualization preceded the program development.

### Needs assessment

Several sources of data, including focus group interviews with IRs, surveys of nursing supervisors who guide triage during RR, feedback from SRs, and verbal feedback received by RR administrative committee members, informed the needs assessment.

The thematic categories identified from the IRs’ perspectives were (1) need for more knowledge about correct use of the RR system, (2) desire for more knowledge and skills to be active participants during a RR event, (3) lack of clarity of the individual roles during a RR, (4) dissatisfaction with self about ‘missing something’ that led to the clinical deterioration, and (5) perceived loss of control over their own patients’ care plan during a RR. The thematic categories identified from the feedback from SRs were that the IRs needed improvement in (1) competency in initial management during a RR, (2) ability to use critical communication, and (3) motivation to participate as a team member in a RR.

### Conceptual framework

After performing a critical review of the literature and engaging in educational expert consultations, we determined that principles posited by the SDT would help address the three main affective consequences of IRs: (1) feelings of loss of control to continue management of their patients, (2) perceived lack of competence in an overwhelming situation, and (3) sense of disconnect from the RR team. The three basic psychological needs – autonomy, competence, and relatedness described in SDT as drivers to foster individuals’ intrinsic motivation and engagement in activities – were applied to our program development.

We proposed that our training program based on SDT and targeted toward IRs would improve the competency and participation of the bedside providers in a RR event. We used SDT as a conceptual framework to guide the development, implementation, and evaluation of the educational activities of the RRT workshop program (Table 1) and explicitly highlighted the three SDT components during the execution of each activity of the workshop.

**Table 1.** Self-determination theory as a conceptual framework to guide the development and implementation of the program.

Self-determination theory components	Why: needs assessment	How: program development and implementation	What: program evaluation
Autonomy	Desire to maintain certain control over care	Highlight opportunities and ways to maintain control over patient’s care during didactic session Encourage exercising autonomy during group case discussions and simulations	Impact on participant perceptions of autonomous patient care and active engagement in activities that allowed for practice of autonomy
Competence	Desire to gain more knowledge and skills	Allow hands-on practice of RR knowledge and skills within a safe learning environment Provide immediate feedback and encouragement to assure individual’s competence	Improvement in clinical and systems knowledge and participants’ perceptions of RR skills
Relatedness	Desire to feel part of the team	Define clearly individual roles within the RR experience Highlight how individuals with variable levels of experience and disciplines can participate and contribute to the team effort Practice teamwork and functioning in a variety of roles	Participants’ engagement in activities that reinforced teamwork and satisfaction with potential of being productively involved with RRs in future

RR: Rapid response.

### Program design

**Figure 1.**
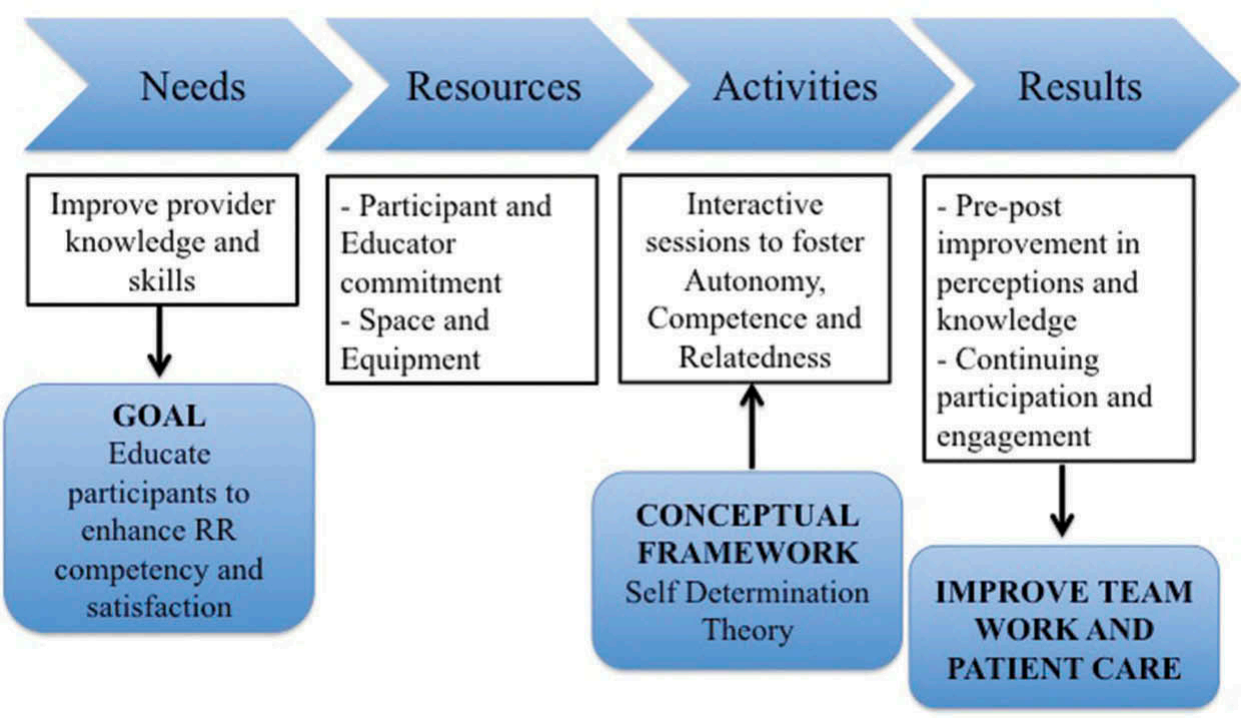
Process map of RRT workshop program development.

The process map used to guide the creation, development, and implementation of the educational program is depicted in Figure 1. The program goals and objectives, as well as the structure of the activities, were discussed and modified with inputs from a focus group composed of leaders (i.e., experts in critical care, nursing managers, residency program leaders, and hospitalist staff) who recognized and subscribed to the utility of this training.

The activities were structured into a 4-h workshop that included multiple instructional methods (interactive didactic, case-based group discussions, skill stations, and simulation). In addition to large-group interactive didactics, the workshop had several small, case-based, group-learning activities through which groups of four to six participants rotated. The workshop organizer intentionally formed these multidisciplinary groups to include participants with diverse levels of experience in each group. The detailed aims and objectives of the individual educational activities are described in Table 2. Several providers in the critical care section, including faculty, fellows, nurses, and advance practice providers, served as the educators and facilitators for the workshop. All the instructors for the simulation activities were providers who had completed structured simulation instructor training provided at our institution.

**Table 2.** Workshop educational activities.

Session	Content	Format	Duration
Interactive didactic	Understand structure of RR system, criteria, and process for RR activation Develop systematic approach to detect and manage common pediatric RR situations	Question and answers with informative slides and videos	30–45 min
Group cases	Practice early detection and response activation Develop communication skills useful for RR situations	Case vignettes practiced by participants grouped into four to six participants per group	1 h
Airway skill station	Learn utility of oxygen delivery devices and airway adjuncts Practice emergent respiratory support	Groups rotated through four skill stations; each group had four to six participants	30 min
Cardiac simulation	Recognize and manage acute cardiovascular instability		30 min
Neuro-respiratory simulation	Recognize and manage acute neurologic and respiratory instability		30 min
Challenging communication station	Learn strategies to navigate challenging or conflicting situations		30 min

RR: Rapid response.

The workshop was advertised through general announcements to hospital-wide pediatric providers, and specific information was disseminated to leaders of the Department of Pediatrics and residency program. Nurses, residents, fellows, faculty, respiratory therapists, and advance practice providers from multiple disciplines within the hospital were invited to participate in the workshop.

### Program evaluation

The program evaluation included (1) *knowledge acquisition*: we developed pre- and posttests with 20 questions each to assess the impact of the workshop training on the participants’ levels of RR-systems knowledge. Clinical case vignettes of commonly occurring pediatric RR events assessed high-order thinking rather than factual knowledge; (2) *SDT components*: we used a reflective self-assessment by participants to determine whether the workshop supported the three components of SDT as it intended. The pre- and posttests included items that address the components of *autonomy* (e.g., pretest question: ‘How frequently do you feel you are able to maintain control over patients care during a RR event?’ Posttest question: ‘Do you feel the RRT workshop has improved your ability to maintain control of your patients care during a RR event?’), *competence* (e.g., pretest question: ‘How frequently do you feel you have the necessary knowledge to actively participate in the management of a decompensating patient?’ Posttest question: ‘Do you feel the RRT workshop has improved your knowledge to actively participate in the management of a decompensating patient?’), and *relatedness* (e.g., pretest question: ‘How frequently do you feel you are able to contribute as a team member in the management of a decompensating patient?’ Posttest question: ‘Do you feel the RRT workshop has improved your ability to contribute as a team member in the management of a decompensating patient?’); (3) *instructional effectiveness*: we used a standard teaching evaluation form recommended for all educational activities at our institution. This 5-point Likert scale questionnaire (ranging from poor to excellent) addressed various learning domains (e.g., organization of the activities, clarity of information, efficacy in improving knowledge and skills, applicability to participants’ practice areas, and time allotment for practice and feedback); (4) *group engagement and performance*: the workshops’ educators and facilitators assessed the participants’ engagement in the learning activities and demonstration of knowledge and skills acquisition during the sessions using a learner-assessment questionnaire.

After completion of the pilot sessions and obtaining preliminary data, we reviewed the feedback received from participants, facilitators, and workshop administrators to identify areas of the program that needed modification.

### Statistics

Descriptive and paired comparative analyses were performed and a significance level of *p* less than 0.05 was used. Statistical computations were carried out using SPSS version 24.0 (IBM Corp. NY, 2016).

## Results

### Preliminary evaluation

During a period of 6 months, we piloted three sessions of the workshop. Forty-two IRs (22 surgical advance practice providers and 20 pediatric residents) participated in the sessions. The average pre-workshop experience level of the IRs in their usual clinical jobs was 1 year (range: 2 months–11 years). The results showed improvement in RR-related systems knowledge for surgical providers and clinical knowledge for both surgical providers and pediatric residents. There was a significant increase in self-reported scores of SDT components: ability to maintain control over the RR situation (a proxy for autonomy pre-mean: 2.12, post-mean: 4.4), capacity to perform competently the tasks needed during RR (a proxy for competence pre-mean: 2.15, post-mean: 4.4), and feeling of inclusion in the team (a proxy for relatedness pre-mean: 2.65, post-mean: 4.5) (Table 3).

**Table 3.** Change in rapid response knowledge and perceptions of self-determination components.

	Surgical advance practice providers			Pediatric residents		
Domains	Pre	Post	*p*-Value	Pre	Post	*p*-Value
Systems knowledge (% accuracy)	75%	96%	*p* < 0.05*	93%	95%	*p* = 0.67
Clinical knowledge (% accuracy)	82%	95%	*p* < 0.05*	80%	95%	*p* < 0.05*
Autonomy (mean score)	2.05	4.3	*p* < 0.01*	2.2	4.5	*p* < 0.01*
Competence (mean score)	2	4.4	*p* < 0.01*	2.3	4.4	*p* < 0.01*
Relatedness (mean score)	2.5	4.5	*p* < 0.01*	2.8	4.5	*p* < 0.01*

*Significance level of *p* < 0.05.

The overall workshop evaluation results are depicted in Table 4. Some of the qualitative narrative of the participants included ‘very useful program,’ ‘positive impact on future RR experience,’ ‘will recommend to peers,’ and ‘would like to have more simulations and refresher sessions through the years.’ The participants’ group evaluations completed by the facilitators showed good engagement in learning and satisfactory acquisition of knowledge and skills during the individual skill stations.

**Table 4.** Program evaluation results (5-point-Likert scale).

Items	Mean score	Standard error	Standard deviation
Workshop organization	4.88	0.05	0.33
Clarity of information	4.83	0.07	0.45
Impact on detection and management of clinical decompensation	4.74	0.08	0.54
Benefit to communication skills	4.67	0.09	0.58
Utility for airway management	4.23	0.13	0.84
Improvement in team participation	4.79	0.07	0.47
Relevance to daily practice	4.74	0.08	0.5
Applicability of knowledge to daily practice	4.54	0.1	0.68
Feedback opportunities	4.46	0.12	0.75

### Program evolution

Several changes have been made to the structure of the workshop, the evaluations, and the organization and maintenance of program-related data, based on the preliminary evaluations and the observations made by educators during workshop sessions.

Modifications made to the program include (1) the length of the didactic session was reduced; (2) group case discussions were structured to cover four main clinical domains: respiratory failure, shock, cardiac failure, and neurologic dysfunction, to address the four most common RR situations; (3) a case bank of clinical vignettes was developed with different scenarios that are applicable to daily practice of various groups of providers and represent the four main domains listed in modification number 2; and (4) the challenging communication station was converted from a discussion exercise to a low-fidelity simulation session to add realistic feeling to the communication challenges. Modifications made to the program evaluations are (1) case vignettes in the pre- and posttests were modified to test clinical knowledge of the four domains of respiratory failure, shock, cardiac failure, and neurologic dysfunction; (2) workshop evaluation forms were simplified (decreased from 25 to 10 questions) to avoid excessive burden of time commitment from the participants (decreased time spent from 15 min to about 5–7 min per participant); (3) five additional questions to assess changes in a participant’s self-efficacy with performance of essential RR tasks, pre- and post-training, were included in the workshop evaluation; and (4) an additional questionnaire was developed to test retention of enhancement in SDT components and self-efficacy at a time remote from the workshop. All questionnaires are being tested for content validity with experts and reliability for SDT components with internal consistency. Modifications made to the data organization are (1) development of a systematic way to record all the responses gathered to allow for optimum paired pre–post testing of each of the questionnaires and (2) creation of a database to record contributions of workshop educators and facilitators with respect to time commitment to provide them with deserved credit.

## Discussion

RR systems are widely implemented across health-care settings and serve multifaceted functions of early detection of clinical instability, prompt response, efficient treatment, and appropriate triage to prevent cardiac arrests [20]. The efficacy of a RR system is invariably dependent on both the response activation and the specialized response components working collaboratively and efficiently [21]. Formal RR training for specialized teams is well established, and evidence of potential benefits of a comprehensive training of all RR providers exists [22,23]. However, minimal literature is available regarding education for bedside providers who activate RR and provide initial response.

### Novelty

We took a novel approach to enhance our RR system by offering training targeted to the IRs, who perform the vital tasks of early detection of clinical instability, response activation, and participation in ongoing care. Additionally to our knowledge, our program is the first of its kind to employ a well-established meta-theory of Self Determination as a conceptual framework for RR education of IRs. SDT has been widely used in guiding educational strategies in health-care settings to achieve adult continued learning and motivation [24]. Throughout our workshop activities, we strategically planned the learning experience and environment to support each of the three components of SDT.

The workshop is unique in the aspect of building multidisciplinary collaborations at both the educators’ and participants’ levels. A variety of providers that included nurses, respiratory therapists, advance practice providers, and physicians committed their time to educate participants. The diversity of experience in the participant groups potentially allowed for realistic experience during simulation scenarios.

### Immediate outcomes

Our program evaluations showed improvement in early educational outcomes. The improved self-assessment related to the components of SDT suggested that the program was on the right track in achieving what it intended to do in terms of supporting learners’ autonomy, competence, and relatedness. The overall positive workshop evaluations and notable engagement in activities suggest that the workshop’s structure and the information provided were considered beneficial and were well received. At the beginning of the program, some apprehension was expressed about voluntary involvement and commitment of participants in the workshop. However, we noted that participants were keen on wanting to enroll and engage in this workshop, which could have been due to their awareness of the need to expand RR-related knowledge.

### Future directions

We plan to continue to enroll participants in the program and gather more data on the merit and worth of the program. We plan to test retention of knowledge and skills and application to real-world situations at a remote time point (6 months–1 year) from the initial training. We will also assess the need for other related educational activities such as refresher sessions for previous participants, multidisciplinary sessions with combined initial responders and SRs as participants, and condensed sessions that will offer specific components of the program for advanced level learners. The diversity and experience level of future participants is anticipated to continually increase. Additionally, the workshop has the potential to address certain RR-related unintended affective consequences that are barriers to effective RR utilization.

### Lessons learned and replication

We determined that certain aspects of program design were critically important in our ability to understand needs of providers that participate in RRs and develop a program that was beneficial and hence well received by participants. These included detailed needs assessment and consultation with educational experts that provided guidance on application of SDT to develop the program. Another important attribute of our engaging workshop was integration of various learning methodologies: didactics, group discussions, simulations, skill stations, etc. We also realized the importance of flexibility with program design, a kind of Educational ‘Plan-Do-Study-Act’ cycle. Our preliminary data have given us several opportunities to perform program modification as described in the “Program evolution” section.

### Limitations

The workshop activities require significant commitment of time and effort from educators and participants, which could impact the sustainability of our program. Given the preliminary nature of the evaluation, we had not performed reliability and validity tests on the questionnaires, and we have included them as a modification for future comprehensive program evaluations. Additionally, our findings on educational outcomes reflect the local context of the program in a single center and may not be directly generalizable. Nonetheless, as we have systematically integrated a theoretical framework into our program design, implementation, and evaluation, we hope that our results can inform others involved in efforts to enhance RR education and quality improvement endeavors. We anticipate the need for ongoing modifications to program structure to address participants and context in diverse settings.

In summary, we successfully developed and implemented a novel educational program guided by SDT to train bedside providers with regards to various aspects of pediatric RR. Our initial findings showed that participants had enhancement in perceived autonomy, competence, and relatedness with regard to RR-related tasks, in addition to improvement in participants’ knowledge and skill sets. We also were able to gather data to guide future modification and improvement of the program.
